# Retraction Note: LNCAROD enhances hepatocellular carcinoma malignancy by activating glycolysis through induction of pyruvate kinase isoform PKM2

**DOI:** 10.1186/s13046-023-02626-z

**Published:** 2023-03-02

**Authors:** Guizhi Jia, Yan Wang, Chengjie Lin, Shihui Lai, Hongliang Dai, Zhiqian Wang, Luo Dai, Huizhao Su, Yanjie Song, Naiwen Zhang, Yukuan Feng, Bo Tang

**Affiliations:** 1 Department of Hepatobiliary Surgery, The First Affiliated Hospital of Guangxi Medical University, 530021 Nanning, Guangxi People’s Republic of China; 2 Key Laboratory of Basic and Clinical Application Research for Hepatobiliary Diseases of Guangxi, 530021 Nanning, Guangxi People’s Republic of China; 3 Department of Pancreatic Cancer, Key Laboratory of Cancer Prevention and Therapy, Tianjin Medical University Cancer Institute and Hospital, National Clinical Research Center for Cancer, 300060 Tianjin, People’s Republic of China; 4 Key Laboratory of Heilongjiang Province for Cancer Prevention and Control, School of Basic Medicine, Mudanjiang Medical University, 157011 Mudanjiang, People’s Republic of China

**Retraction Note: J Exp Clin Cancer Res 40, 299 (2021)**

**https://doi.org/10.1186/s13046-021-02090-7**

The Editor in Chief has retracted this article. After publication, concerns were raised about an apparent partial overlap between the leftmost SNU-449 migration panel in Fig. 2C and the top LNCAROD+2-DG panel in Fig. 4G. The authors were unable to provide a satisfactory set of raw data on request. Additionally, the issues regarding the provenance of cell lines were not completely resolved in the published version of the article. The editor, therefore, has lost confidence in the integrity of the findings of this article. The authors have not explicitly stated that they agree to this retraction.

